# Impulsivity and socio-demographic variables among bipolar disorder patients

**DOI:** 10.1192/j.eurpsy.2021.526

**Published:** 2021-08-13

**Authors:** S. Ajmi, S. Najjar, I. Baati, J. Masmoudi

**Affiliations:** 1 Psychiatrie A, hospital University Hedi Chaker, sfax, Tunisia; 2 Psychiatry A, hedi chaker hospital, Sfax, Tunisia; 3 Psychiatrie “a” Department, Hedi Chaker Hospital University -Sfax - Tunisia, sfax, Tunisia

**Keywords:** Impulsivity, sociodemographic, biopolar disorder

## Abstract

**Introduction:**

Impulsivity is a psychiatric symptom that seems to be more prevalent in some mental disorders such as bipolar disorders (BD). It is a trait that seems to be influenced by many socio-demographic variables across BD.

**Objectives:**

The aim of our study is to examine the relationship between impulsivity and these variables.

**Methods:**

We performed a cross sectional study on 30 patients diagnosed with BD and consulting at the Psychiatric department of Hedi Chaker Hospital. Patients were euthymic during the time of the study confirmed by administration Young Mania Rating Scale (YMRS) and Montgomery Depression Rating Scale (MDRS). The socio-demographic data was obtained. Impulsivity was evaluated using the Barratt Impulsiveness Scale (BIS-11)

**Results:**

The study sample consisted of 30 patients (10 men and 20 women). The mean age of the sample was 45.83 years (SD= 11. 63). Seventeen patients (56.7%) were married. More than half of the subjects (76.7%) were unemployed and 26.7% were not educated. Of the studied patients 83.3% were drug free, 43.3% were smoker and 16.7% were alcoholic. The mean BIS11 score was 75. 60 (SD=5.51) and 76.7% had a high level of impulsivity. No correlation was found between the level of impulsivity (BIS-11 scores) and age, gender, marital status, being a current smoker, using drug or alcohol or job status (p=0.082; p=0.760; p=0.087: p=0.977; p=0.847; p=0.708).

**Conclusions:**

Further studies should be realized to fully characterize impulsivity in BD and, therefore, make it a target for future therapeutic models.

